# The power of calmness in times of the COVID-19 pandemic: The different roles of peace of mind and career calling in enhancing work engagement—A mediation analysis based on social support

**DOI:** 10.3389/fpsyg.2023.1104430

**Published:** 2023-03-09

**Authors:** Yiheng Xi, Li Zhou, Dong Guo

**Affiliations:** ^1^School of Public Policy and Management, University of Chinese Academy of Sciences, Beijing, China; ^2^Faculty of Education, The University of Hong Kong, Pokfulam, Hong Kong SAR, China

**Keywords:** the COVID-19 pandemic, peace of mind, social support, career calling, work engagement, conversation of resource theory

## Abstract

**Introduction:**

The prevention and control of the COVID-19 pandemic has taken on a “New Normal” form, which necessitates a calm and peaceful social mentality. This study delves into the Chinese socioculturally oriented emotion construct of peace of mind (PoM) with regard to how it may affect employees’ work engagement in times of the pandemic. Based on the conversation of resource (COR) theory, we develop a model in which the relationship between PoM (i.e., a low-arousal positive affective state) and work engagement and the relationship between career calling (i.e., a high-arousal positive state) and work engagement are both mediated by social support.

**Methods:**

A total of 292 employees from 18 companies in Wuxi and Dalian, China, were surveyed at two different time points during the COVID-19 pandemic.

**Results:**

The results show that both relationships were mediated by social support; furthermore, after the mediating effect of social support on the relationship between PoM and work engagement was controlled for, the relationship between career calling and social support failed to reach significance.

**Discussion:**

The findings attest to the unique advantages of PoM in boosting employees’ resource conservation and interpersonal communication in public crises. Possible implications on applying the incentive mechanism of PoM in the workplace are discussed.

## 1. Introduction

The ongoing COVID-19 pandemic has raised a growing public health concern worldwide in both physical and psychological aspects. Prolonged lockdown and quarantine have turned people’s life upside down, which has given rise to a dramatic decline in social mentality. Recent research by positive psychologists has explicitly pointed to the critical importance of fostering individuals’ subjective well-being (SWB) and building a positive social mentality, which is especially true in times of major public crisis, such as the COVID-19 pandemic.

In a time when the prevention and control of the pandemic has become a New Normal^1^ around the globe, companies are making efforts to accelerate work and production resumption. Building a positive public mentality thus becomes necessary. Studies have established that SWB can serve as a major stimulator of employees’ engagement at workplace (Kim et al., 2018). However, a major challenge faced by researchers is that SWB is not only biologically determined, but also critically affected by social and cultural orientations (Turner and Stets, 2005; Kawahara et al., 2021). Specifically, SWB has traditionally been conceptualized as the presence of positive affect (PA), the absence of negative affect (NA), and the cognitive components of life satisfaction (Ryan and Deci, 2001). SWB is indicative of experiencing PA to the greatest degree and NA to the lowest degree, echoing the hedonic view of happiness valued in western or individualist societies (Diener, 2000, 2012). A smaller but still substantial niche of researchers have indicated that the conception of individual well-being in eastern cultures may be a far cry from the hedonic view of happiness developed in western cultures (Kampfe and Mitte, 2009; Joshanloo, 2014; Disabato et al., 2015).

Drawing from Chinese cultural tradition, Lee et al. (2013) developed a positive emotion construct, *peace of mind* (PoM), to describe the distinctive affective well-being valued in Chinese culture. PoM refers to an affective state of inner peace and internal harmony. Inner peace captures the states governed by low-arousal positive (LAP) affects, such as peacefulness, calmness, and serenity, which occur when individuals are relieved of both the urge to evade negativeness and the desire to pursue positiveness. Internal harmony captures the state of harmony and balance, which refers to the process of self-control which can help individuals deal with feelings that are excessively intense, such as anger, sorrow, and enthusiasm, and ensure their emotional state fluctuates within a moderate range. The two aspects of PoM are intertwined such that individuals can either attain inner peace through the process of harmony or achieve harmony *via* maintaining a peaceful state of mind, thus constituting a coherent emotional state (Lee et al., 2013). The socioculturally oriented conception of inner peace and harmony has been embedded in traditional Chinese philosophy, including the principle of equilibrium (*zhong*) in Confucianism, the balance between *Yin* and *Yang* (opposite forces) advocated by Taoism, and the state of cessation of desire in Buddhism (Lee et al., 2013). The present study aims to investigate the low-arousal emotion of PoM in the Chinese workplace context with respect to how it may affect employees’ work engagement in times of the COVID-19 pandemic, in contrast to high-arousal positive (HAP) emotions.

## 2. Theory and hypotheses

### 2.1. PoM and work engagement

PoM, as a positive affective state (Lee et al., 2013), is claimed to be a unique psychological resource with stimulating functions. PoM involves the general characteristics of a positive emotion. Unlike negative emotions associated with a specific action tendency (e.g., anger leads to aggressive behaviors), PoM, as a positive emotion, activates normalized or generalized action tendencies (Fredrickson and Branigan, 2005). Studies have showed that positive emotions are able to induce environment-related action tendencies (Guo and Wang, 2007). Individuals in the grip of positive emotions in the workplace are more likely to feel a compulsion toward work, which can be interpreted as work engagement.

Furthermore, PoM contributes to developing personal resources, including intrapersonal resources and interpersonal resources. As a positive emotion, PoM broadens individuals’ momentary thought-action repertoires (Fredrickson and Branigan, 2005). Specifically, PoM broadens individuals’ scopes of attention and cognition and makes more information available for cognitive processing, so that more intrapersonal resources can be put into work (Anjum et al., 2014; Yu et al., 2019). Besides, PoM builds interpersonal resources. PoM allows for strong and extensive social connections and provides an impetus for all activities that involve “people” (Fredrickson, 2003). The affluence of work resources is necessary for stimulating individuals’ work engagement.

Moreover, PoM is conducive to resource conversation. PoM differentiates itself from other positive emotions for its emphasis on the culture-specific state of internal harmony, which reflects people’s expectation for a non-conflicting group-based social relationship system. In the Chinese society, direct confrontation in human interaction is generally interpreted as a threat or even an infringement (Friedman et al., 2006). Individuals with PoM are more likely to maintain a balanced affective state (Lee et al., 2013), avoid conflicts, turn inward and inspect themselves and behave in a coordinated way. Therefore, their resources are conserved through nurturing a balanced and stable state (or a low-arousal affective state) in which less resources are to be consumed compared to an intense state (or a HAP state). Individuals governed by PoM may thus have better access to work resources, a primary incentive for improving work engagement. That is, PoM may be an important antecedent of work engagement. Hypothesis 1 is proposed:

*H1*: PoM is positively correlated with work engagement.

### 2.2. Career calling and work engagement

Career calling is perceived as a meaningful passion (Bunderson and Thompson, 2009), which emphasizes working for the benefit of the community and regarding this altruistic orientation as the meaning of life (Gu et al., 2018). Individuals with career calling approach their work with a strong sense of desire and push for the fulfillment of goals or tasks. Evidently, this sense of passion will lead to increased individual motivation and bring impetus to work (Sosnowska et al., 2017). The close relation between career calling and work engagement has been confirmed in employee motivation research (Hirschi and Herrmann, 2013; Xie et al., 2016).

Previous studies on the relationship between career calling and work engagement have incorporated the view of the meaning of life (Xie et al., 2016). Specifically, career calling is a perceived feeling that endows individuals with a sense of meaning and identity at work (Dik and Duffy, 2009), wherein individuals gain psychological satisfaction, motivation, and work resources. Bunderson and Thompson’s (2009) study on the meaning of work found that employees who perceive their work with a calling feel happiness and satisfaction even if they are engaged in low-paid jobs. To sum up, individuals with career calling have more attention, vitality and devotion at work, which, in turn, lead to increased employees’ motivation. Therefore, career calling may be an important factor that influences employees’ work engagement. Therefore, hypothesis 2 is formulated:

*H2*: Career calling is positively correlated with work engagement.

### 2.3. The mediating role of social support

Social support refers to emotional or instrumental assistance that contributes to individuals’ goal achievement, such as counseling, care, and opportunity (Hobfoll, 2009). Social support helps individuals to build interpersonal relationships and is considered a universally valuable resource (Benoit and Ditommaso, 2020). According to the COR theory, individuals have strategic motivations to create, accumulate, and protect valuable resources (Halbesleben et al., 2014). Social support is a relational resource which people create and protect out of strategic considerations (Wilson et al., 2020). Previous studies found that people tend to provide social support to others with the purpose of accumulating resources for themselves (Hobfoll, 2002). Empirical evidence also suggested that people are prone to make social investment in those who are likely to achieve value maximization (Gibbons, 2004). Therefore, individuals endowed with resource advantages could be more likely to obtain social support.

As previously discussed, individuals governed by either PoM or career calling have resource advantages. Studies showed that individuals are apt to make resource investment in those who possess resource advantages (Gibbons, 2004). Therefore, individuals with either PoM or career calling tend to obtain social support from others. Besides, individuals in possession of valued resources are treated more favorably or “with a lower set of standards” (Hobfoll, 2002, p. 319), which allows them to benefit from positive interpersonal communication. Therefore, both PoM and career calling are related to increased social support.

Additionally, social support promotes work engagement. Hobfoll (2002) proposed that resources that boost work engagement include both relational resources and individual resources. Relational resources refer to resources that exist in social interactions and are independent of individuals themselves. Evidently, social support is a kind of relational resource, which exists in organizations and helps employees fulfill job-related tasks, reduce psychological strain, and attain work engagement. (Korunka et al., 2009; Xi et al., 2020).

To sum up, individuals driven by PoM and career calling have better access to social support. Social support, in turn, leads to increased work engagement. Therefore, we propose that social support mediates the relationships both between PoM and work engagement and between career calling and work engagement. Hypothesis 3 is thus proposed:

*H3*: Social support mediates the relationship between PoM and work engagement.

### 2.4. Differences in the mediating effects of social support

As discussed before, individuals with either PoM or career calling are more likely to obtain social support from others. This is because people have the need for resource accumulation and are thus willing to build relationships with and make resource investment in those better endowed with resources. However, this only happens when their existing resources are not lost or threatened with loss. The COR theory posits that resource conservation always takes priority (Hobfoll, 2002). People are motivated to accumulate resources as a means of lowering the risk of resource loss. In other words, once resource loss occurs, people will no longer be particularly interested in making resource investment in others (Zeidner et al., 2011).

Compared to PoM, HAP emotions are more likely to cause resource loss (Fredrickson, 2003). Once individuals with HAP emotions fail to obtain resources that meet their high expectations, they may experience social and psychological liabilities, as a result of which stressors are generated. They will then make further resource investment in a bid to make up for failings of resource acquisition or acquired resources that fall short of their expectations. In this process, individual resources (e.g., attention) are preempted by the increasing psychological stress generated. This may lead to a resource loss spiral mentioned in the COR theory. That is, individuals who experienced resource loss and are eager to stop or make up for the lost resources may end up losing more (Natascha et al., 2021). The more emotionally intense they are, the more likely they are to suffer from further resource loss.

Additionally, when individuals are threatened with resource loss, it becomes difficult to for them present goodwill in interpersonal communication. In Chinese culture, intense emotional release is normally interpreted as a threat (Friedman et al., 2006). By contrast, individuals governed by PoM may find themselves in a more stable and well-regulated emotional state through self-control and thus feel neither overly excited nor upset (Yu et al., 2019). Besides, they have access to resources just as much as they need or expect, instead of seizing too many resources, so that they are likely to be treated more favorably by others (Yu et al., 2019). Therefore, individuals with PoM have better access to emotional and instrumental assistance from others, such as counseling, care, and opportunity, which are collectively referred to as social support (Hobfoll, 2009).

Career calling as a self-driving force is marked by enthusiasm or passion. Studies have identified career calling as an important motivational antecedent of work engagement that provides short-term incentives. However, in a relationship-based society like China, individuals who over-emphasize personal fulfillment may be treated less favorably. As a result, they may be less likely to obtain resource investment from others and benefit from positive relational resources. Career calling is not considered as a stable affective state, compared to PoM. The strong passion aroused by calling may result in excessive use of individual resources or resources threatened with loss (Szymaniak and Zajenkowski, 2021). Individuals with career calling may not obtain resource advantages and produce work engagement through the accumulation of relational resource (i.e., social support) as much as those with PoM. Therefore, we propose that social support may have a stronger mediating effect on the relationship between PoM and work engagement than on the relationship between career calling and work engagement. The following hypothesis is thus formulated:

*H4*: Social support does not mediate the relationship between career calling and work engagement as much as it does to the relationship between PoM and work engagement.

**Figure 1:**
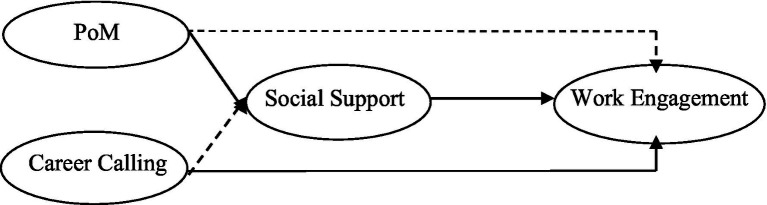
Hypothesized research model.

The hypothesized research model is presented in Figure 1.

## 3. Materials and methods

### 3.1. Participants and data collection

This study administered a questionnaire survey in 300 Chinese employees from 18 companies in Wuxi and Dalian, China. Data were collected in different time points during the production resumption period in China after the pandemic had largely been brought under control, where employees were allowed to return to the workplace and no longer worked from home. Located in the southeast of China, Wuxi is part of the Yangtze River Delta Economic Zone which has a high level of economic development. Wuxi is also a demonstration city for scientific and technological innovation and industrial restructuring. Due to the proper prevention and control measures, the impact of the pandemic on the economic operation and people’s lives in Wuxi was relatively small. Dalian, located in northeast China, is a typical old industrial city. For many years, Dalian has been faced with difficulties in economic development, such as backward industry and failure of industrial transformation. Meanwhile, Dalian is an important harbor city in which frequent cold chain transportation has repeatedly caused the outbreak of the pandemic. This has seriously affected the resumption of work and production in the city. Based on these, we chose Wuxi and Dalian which are in stark contrast in terms of economic and technological development and the degree to which they have been affected by the pandemic.

We took 1 month as the time interval between the two surveys. Both surveys were administered using a composite online questionnaire. The link of the questionnaire survey was sent to the supervisors or human resource managers of the 18 companies who forwarded it to their employees *via* email or WeChat, a Chinese social media. The first survey collected information on participants’ demographics and experiences regarding PoM and career calling. The second survey conducted 1 month later elicited information concerning social support and work engagement. A total of 300 questionnaires were collected. 292 valid responses were finally used after matching with samples gathered in the first survey, with an effective rate at 97.33%. Male employees accounted for 53.15% of the samples, and female employees 46.85%, with a mean age of 30.19. 226 participants held a college degree or above. The years of working of the participants ranged from 1 to 28.

### 3.2. Research instruments

*Social support*. A simplified version of the Questionnaire on the Experience and Evaluation of Work (QEEW) was used to measure social support (Van Veldhoven and Meijman, 1994), which was based on a 7-point Likert scale. The consistency coefficient was 0.83.

*Peace of mind.* The 7-item PoM scale developed by Lee et al. (2013) was used to measure PoM based a 5-point Likert scale. The alpha reliability was 0.84.

*Career calling.* The “presence of calling” section of Calling and Vocation Questionnaire (CVQ) scale (Dik et al., 2012) was used to measure career calling. It included 3 sub-scales: transcendent summons, purposeful work, and pro-social orientation, the consistency reliability of which was 0.89, 0.79, and 0.85, respectively. The reliability of the whole scale was satisfactory (0.84).

*Work engagement.* A simplified version of the Utrecht Work Engagement Scale (UWES) was used to measure work engagement, which consisted of nine items. Three dimensions were included: vigor, dedication, and absorption (Zhang and Gan, 2005). The consistency coefficient was 0.93 for the whole scale, 0.78, 0.84, and 0.83 for the three dimensions, respectively.

## 4. Results

### 4.1. Descriptive analyses

Table 1 reported the means, variances, reliabilities, and correlations of the measured variables. The zero-order correlations of all the four variables reached significance (*p* < 0.01). Besides, PoM was significantly correlated with social support and work engagement, both with a significance level of 0.001. Career calling was also significantly correlated with work engagement (*p < 0*.001) and social support (*p* < 0.01).

**Table 1:** The means, standard deviations (SDs), correlations and consistency coefficients of the variables.

	Mean	SD	1	2	3	4
1. PoM	3.44	0.70	0.84			
2. Social support	3.99	1.11	0.24^***^	0.83		
3. Career calling	2.61	0.53	0.24^***^	0.16^**^	0.84	
4. Work engagement	3.27	1.14	0.36^***^	0.19^**^	0.62^***^	0.93

*N* = 292; ^***^p < 0.001, ^**^p < 0.01, ^*^p < 0.05; Consistency coefficients are on the diagonal.

### 4.2. Discriminant validity test

Confirmatory factor analyzes were run to examine the discriminant validity of the four measured variables before any further analysis. Four hypothesized competitive models were proposed accordingly, including 3 three-factor models and 1 four-factor model. As shown in Table 2, the fit of the four-factor model was superior to that of the three-factor models (*χ*^2^/df = 2.009, *RMSEA* = 0.062, *CFI* = 0.912, *TLI* = 0.901, *SRMR* = 0.060). Besides, we compared the four-factor model and the best-fit three-factor model, which suggested that the difference of fit between these two models reached significance (*χ*^2^/df = 175.897). Therefore, the four variables studied were well discriminable.

**Table 2:** Results of discriminant validity analysis.

Variables	*χ*^2^/df	*χ*^2^/df	RMSEA	CFI	TLI	SRMR
POM + CALL + SS + WE	6.637	6.637	0.147	0.500	0.453	0.129
POM, CALL + SS + WE	5.963	195.46	0.138	0.562	0.518	0.124
POM, CALL, SS + WE	4.339	208.06	0.113	0.708	0.676	0.105
POM, CALL, SS, WE	2.009	175.897	0.062	0.912	0.901	0.060

*N* = 292; PoM, peace of mind; CALL, career calling; SS, social support; and WE, work engagement.

#### 4.2.1. Primary effect test

**Figure 2:**
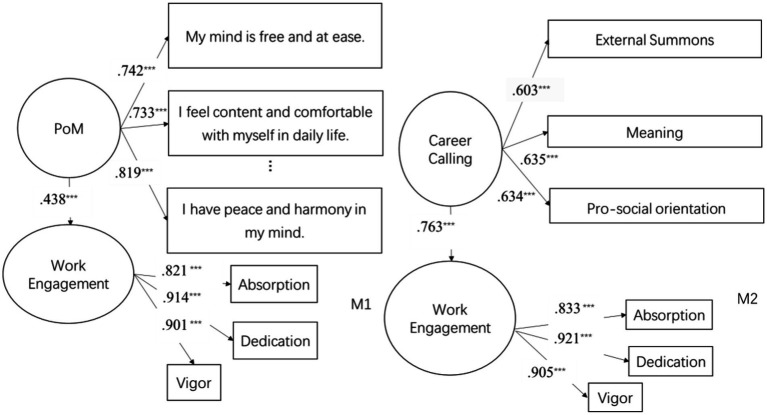
Primary effects analysis.

Structural equation modeling (SEM) was used to test the predictive effects of PoM and career calling on work engagement. According to their factor structure, PoM was set as a single-factor latent variable with all the items as its observed variables, career calling as a latent variable with three observed variables (external summons, meaningful orientation, pro-social orientation), and work engagement as a latent variable with three observed variables (vigor, dedication, absorption). As indicated in Figure 2, both mediation models had a good fit (M1: *χ*^2^/df = 2.687, *p* < 0.001; *RMSEA* = 0.077; *CFI* = 0.970; *TLI* = 0.957; *SRMR* = 0.046; M2: *χ*^2^/df = 2.263, *p* < 0.001; *RMSEA* = 0.068; *CFI* = 0.944; *TLI* = 0.932; *SRMR* = 0.045). The predictive effects of PoM (*t* = 0.438, *p* < 0.001) and career calling (*t* = 0.763, *p* < 0.001) on work engagement both reached significance. H1 and H2 were thus validated.

#### 4.2.2. Mediating effect test

**Figure 3:**
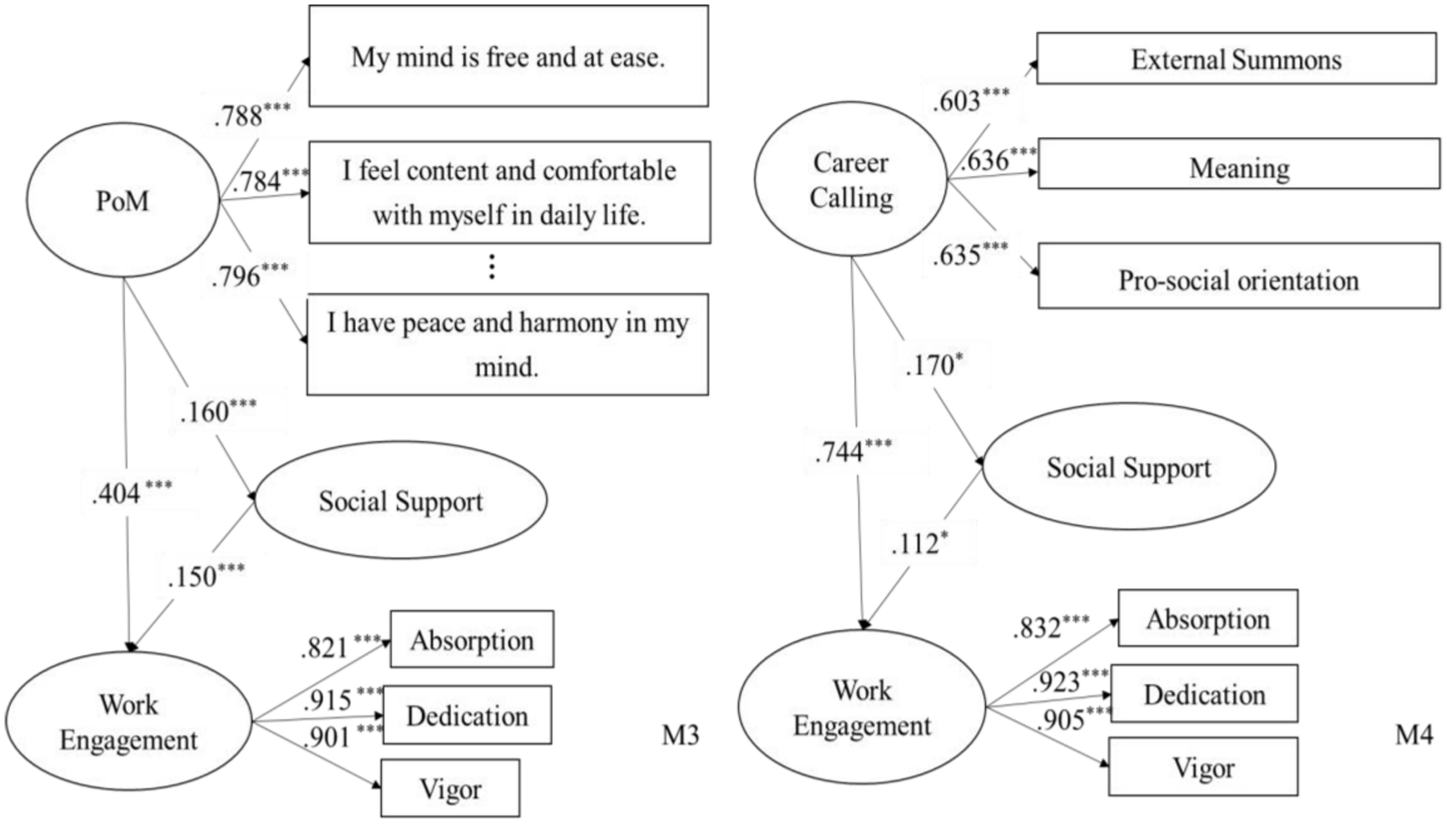
Analysis of the mediating effects of social support.

SEM was used again to test the mediating effect of social support on the relationships between PoM and work engagement and between career calling and work engagement. Two models were hypothesized based on the theoretical assumptions. The cutoff criterion for the goodness of fit, including *RMSEA* and *SRMR* ≤ 0.08, *CFI,* and *TLI* ≥ 0.95, were adopted (Hu and Bentler, 1999). As shown in Figure 3, both models had a good fit (M3: *χ*^2^/df = 2.072, *p* < 0.001, *RMSEA* = 0.062, *CFI* = 0.964, *TLI* = 0.955, *SRMR* = 0.047; M4: *χ*^2^/df = 1.873, *p* < 0.001, *RMSEA* = 0.056, *CFI* = 0.948, *TLI* = 0.939, *SRMR* = 0.044).

As shown in the Figure 3, Model 3 showed that PoM had a significant positive effect on social support (*t* = 0.160, *p* < 0.05), and social support had a significant positive effect on work engagement (*t* = 0.112, *p* < 0.05). Besides, Model 4 showed that career calling had a significant positive effect on social support (*t* = 0.170, *p* < 0.05), and social support had a significant positive effect on work engagement (*t* = 0.112, *p* < 0.05).

Moreover, we used Bootstrap, the re-sampling technique, to verify the indirect effects of PoM and career calling on work engagement through social support (Baron and Kenny, 1986). 500 samples were drawn repeatedly from the original dataset, the results of which showed that the mediating effect of social support on the relationship between PoM and work engagement had an effect size of 0.035 [0.012, 0.083], and the mediating effect of social support on the relationship between PoM and work engagement had an effect size of 0.034 [0.027, 0.072]. In other words, social support mediated the relationship between PoM and work engagement and the relationship between career calling and work engagement, separately. H3 was thus validated.

#### 4.2.3. A comparison of the mediating effects

**Figure 4:**
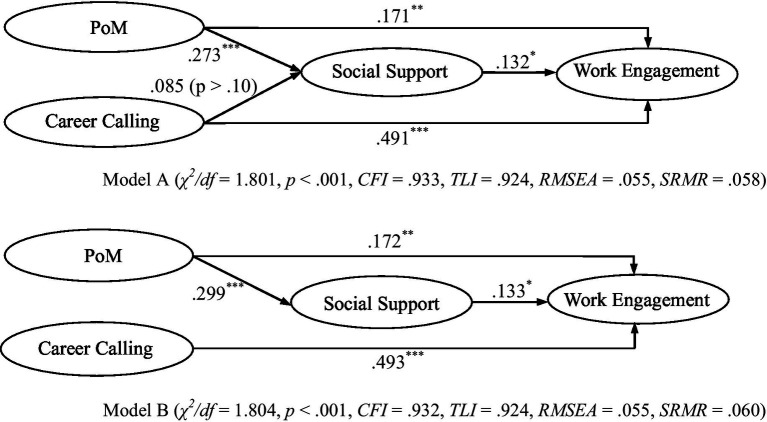
Structural equation model of the latent variables (Model A vs. Model B). Model A (*χ*^2^/df = 1.801, *p* < 0.001, *CFI* = 0.933, *TLI* = 0.924, *RMSEA* = 0.055, *SRMR* = 0.058). Model B (*χ*^2^/df = 1.804, *p* < 0.001, *CFI* = 0.932, *TLI* = 0.924, *RMSEA* = 0.055, *SRMR* = 0.060). *N* = 292; ^***^p < 0.001, ^**^p < 0.01, ^*^p < 0.05.

The results reported above verified the hypotheses that social support mediated the effects of both PoM and career calling on work engagement. Considering the coexistence of the two mediation paths, we further proposed a model (Model A) which included all the variables and paths observed. Model A indicated a good fit (*χ*^2^/df = 1.801, *p* < 0.001, *CFI* = 0.933, *TLI* = 0.924, *RMSEA* = 0.055, *SRMR* = 0.058). As shown in Figure 4, social support was able to mediate the effect of PoM on work engagement but failed to mediate the effect of career calling on work engagement.

Another model (Model B) was proposed in which the direct path from career calling to work engagement was removed. We compared Model B (*χ*^2^/df = 1.804, *p* < 0.001, *CFI* = 0.932, *TLI* = 0.924, *RMSEA* = 0.055, *SRMR* = 0.060) with Model A in terms of goodness of fit, which showed that Model B did not have a significantly better fit than Model A (*χ*^2^/df = 2.38, *p* > 0.05). This indicated that on the basis of parsimony, Model B was a better fit for the data.

As shown in Figure 4, the path coefficients between PoM and work engagement (*b* = 0.171, *p* < 0.01) and between career calling and work engagement (*b* = 0.491, *p* < 0.001) was both significant. Meanwhile, we analyzed the mediating effects of social support on the relationships between PoM and work engagement and between career calling and work engagement. It showed that social support mediated the relationship between PoM and work engagement, but failed to mediate the relationship between career calling and work engagement. Furthermore, because Model B proved to be the model with the best fit, the removal of the path from career calling to social support was justified. In other words, the mediating effect of social support on the relationship between career calling and work engagement in the model was not supported.

Moreover, we used multivariate delta method to evaluate the difference between the two mediation paths. As presented in Table 3, the mediating effect of social support on PoM and work engagement was more significant than that on career calling and work engagement (*diff* = 0.049, *p* < 0.05). In other words, social support had a stronger mediating effect on the relationship between PoM and work engagement than on the relationship between career calling and work engagement. Therefore, H4 was verified.

**Table 3:** Results of the comparison between the two mediating effects.

Path	Total effect		Indirect effect		Direct effect	
	B 95% CI		B 95% CI		B 95% CI	
CALL → SSL → WE	0.410^***^	[0.313, 0.507]	0.009	[−0.007, 0.025]	0.401^***^	[0.301, 0.501]
PoM → SS → WE	0.332^***^	[0.177, 0.486]	0.058^*^	[0.009, 0.106]	0.274^**^	[0.116, 0.432]
Difference	0.078	[−0.129, 0.285]	−0.049^*^	[−0.093, −0.005]	0.127	[−0.081, 0.335]

*N* = 292; ^***^p < 0.001, ^**^p < 0.01, ^*^p < 0.05; PoM, Peace of mind; CALL, career calling; SS, social support; and WE, work engagement.

## 5. Discussion

The present study investigated PoM (a LAP affective state) and career calling (a HAP affective state) and their influences on employees’ work engagement in times of the COVID-19 pandemic. The results verified the assumption that both PoM and career calling indirectly affected work engagement through social support. More importantly, employees with PoM have better access to social support and then benefit from more work engagement, compared to those with career calling. It was suggested that PoM is a valuable psychological resource with unique advantages in stimulating employees’ engagement in the workplace in times of major public crises, such as the COVID-19 pandemic.

### 5.1. Theoretical implications

Firstly, the present study attempted to examine the regional heterogeneity of social public mentality faced with major public crises by incorporating two different Chinese cities, Wuxi and Dalian, in times of work and production resumption immediately after the COVID-19 pandemic was largely brought under control in China. Wuxi is in the southeast of China, whereas Dalian is in the northeast of China. The differences in geographical location, natural climate, history and culture may lead to differences in individuals’ perceptions toward subjective wellbeing and work engagement. Besides, Wuxi is part of the Yangtze River Delta Economic Zone, which has a high level of economic development. It is also a demonstration city for scientific and technological innovation and industrial restructuring. By contrast, Dalian is a typical old industrial city in China and has long been faced with difficulties in economic development, including backward industry and failure of industrial transformation. In addition, owing to proper prevention and control measures, Wuxi has suffered less from the impact of the pandemic on its economic operation and people’s daily life. In contrast, Dalian is an important harbor city in which frequent cold chain transportation has repeatedly caused the outbreak of the pandemic. This has seriously affected the resumption of work and production in the city. Therefore, regional heterogeneity was anticipated in social public mentality. However, no significant differences were found. Analysis on either city led to the same results with what was reported in this study. We thus assume that the COVID-19 pandemic is a global challenge and has created an overall social mentality that may not necessarily vary from city to city. In addition, the stimulating effect of PoM, which was particularly attended to in this study, may not be influenced by economic or cultural disparities, but instead, has universal significance.

Secondly, this study verified the construct of PoM and the validity of the PoM scale during the COVID-19 pandemic. PoM is gaining increasing attention in psychology and medicine research in recent years. However, as a typical emotional state experienced in the workplace, PoM has largely been neglected in organizational behavior research. This study filled one of the gaps by examining the characteristics of PoM and its unique advantages in boosting employees’ engagement in times of major health crises that causes mass panic.

Thirdly, this study lent support to the previously established view that eastern conceptualization of subjective well-being is fundamentally different from the high-arousal affective states valued in Western cultures (Lee et al., 2013). Uchida and Ogihara (2012) pointed out that in individualist societies people tend to hold a hedonic perception of happiness, while in collectivist societies people show favoritism to a relationship-oriented perception of happiness. Therefore, individual happiness or well-being developed in the Chinese cultural context may help individuals build more harmonious interpersonal relationships, which has not been examined in previous studies on PoM. In the present study, we differentiated the influencing mechanism of PoM on work engagement from that of high-arousal affects (i.e., career calling) through comparing the mediating effects of social support on PoM and work engagement and on career calling and work engagement. It revealed the positive effect of PoM in gaining social support and boosting interpersonal communication. This finding corroborated previous studies that claimed that PoM have unique advantages in building and maintaining personal resources (Yu et al., 2019; Zhou et al., 2021).

Finally, this study extended the scope of the COR theory by incorporating the perspective of relational resources (Hobfoll et al., 2018). Previous studies have predominantly focused on the conservation of intrapersonal resources, while few have taken account of interpersonal resources. This study demonstrated that social support, as a type of relational or interpersonal resources, is also in line with resource accumulation and investment principles involved in the COR theory.

### 5.2. Practical implications

This study should make an important contribution to the incentive effect of PoM in human resource management. Chinese employees seem to have always been encouraged to be “full of beans” or keep themselves in a HAP affective state, so as to increase motivation and work engagement. HAP affects also predominate in positive psychology research. However, it is argued that the positive emotional state valued in Chinese cultural context is marked by reserved low-arousal affects, rather than emotionally charged high-arousal affects. LAP affects not only ensure that individuals maintain a peaceful and stable state, but also contribute to favorable interpersonal communication. Individuals governed by LAP affects are also able to relieve themselves of the fears that “tall trees catch much wind.” Meanwhile, Chinese enterprises should not overemphasize evoking employees’ HAP emotions as a means of stimulating work engagement. Instead, employees should be encouraged to keep a calm and peaceful mind through self-control and self-discipline.

Finally, this study is situated in the context of the COVID-19 pandemic by which the physical and mental health of the public was severely impaired. The eruption of the pandemic and the accompanying prevention and control measures, including mass quarantine, social distancing, have turned the public life upside down. However, as the prevention and control of the pandemic has gradually taken on a new normal form, the resumption of enterprise activities and employees’ work engagement has become a major concern, which is also the primary research question addressed in this study. The study revealed that PoM has resource advantages in terms of both intrapersonal and interpersonal resources, which makes work resources available to employees facing major crises. Therefore, enterprises should be aware of the importance of building and maintaining PoM in boosting employees’ work engagement.

### 5.3. Limitations and prospects

Several limitations to this exploratory study should be addressed in future research. First, since the measures used in this study were based on employees’ self-ratings, their cognitive biases may interfere with the accuracy of the results. Future research could use peer appraisal to elicit more objective data. Second, future work is needed to explore the mechanisms and boundary conditions of the effect of PoM on work engagement. For example, emotional stability could be an important mechanism underlying the impact of PoM on work engagement. Finally, this study included only Chinese samples, without examining the individual difference of PoM in different countries or contexts. As Lee et al. (2013) suggested, PoM is more valued in collectivist cultures, so its role in the workplace might vary across cultures. More studies from the perspective of cultural variation are required.

## Data availability statement

The raw data supporting the conclusions of this article will be made available by the authors, without undue reservation.

## Ethics statement

The studies involving human participants were reviewed and approved by Academic Ethics Committee of Renmin University of China. The patients/participants provided their written informed consent to participate in this study.

## Author contributions

YX: conceptualization, methodology, writing and original draft preparation, and formal Analysis. LZ: investigation, validation, and visualization. DG: conceptualization, review & editing, and project administration. All authors contributed to the article and approved the submitted version.

## Conflict of interest

The authors declare that the research was conducted in the absence of any commercial or financial relationships that could be construed as a potential conflict of interest.

## Publisher’s note

All claims expressed in this article are solely those of the authors and do not necessarily represent those of their affiliated organizations, or those of the publisher, the editors and the reviewers. Any product that may be evaluated in this article, or claim that may be made by its manufacturer, is not guaranteed or endorsed by the publisher.

## Footnotes

^1^"New Normal" is a management form adopted by the Chinese government for the prevention and control of the COVID-19 pandemic, which denotes that the pandemic is likely to be prolonged and thus authorities and citizens may treat its prevention and control as a long-term task.
